# Activation of Matrix Hyaluronan-Mediated CD44 Signaling, Epigenetic Regulation and Chemoresistance in Head and Neck Cancer Stem Cells

**DOI:** 10.3390/ijms18091849

**Published:** 2017-08-24

**Authors:** Lilly Y. W. Bourguignon, Christine Earle, Marisa Shiina

**Affiliations:** San Francisco Veterans Affairs Medical Center and Department of Medicine, University of California at San Francisco & Endocrine Unit (111N2), 4150 Clement Street, San Francisco, CA 94121, USA; cearle777@gmail.com (C.E.); marishiina@gmail.com (M.S.)

**Keywords:** cancer stem cells (CSCs), hyaluronan (HA), CD44, miR-10b, DOT1L, epigenetic regulation, HNSCC

## Abstract

Head and neck squamous cell carcinoma (HNSCC) is a solid tumor composed by a genotypically and phenotypically heterogeneous population of neoplastic cells types. High recurrence rate and regional metastases lead to major morbidity and mortality. Recently, many studies have focused on cellular and molecular mechanisms of tumor progression that can help to predict prognosis and to choose the best therapeutic approach for HNSCC patients. Hyaluronan (HA), an important glycosaminoglycan component of the extracellular matrix (ECM), and its major cell surface receptor, CD44, have been suggested to be important cellular mediators influencing tumor progression and treatment resistance in head and neck cancer. HNSCC contains a small subpopulation of cells that exhibit a hallmark of CD44-expressing cancer stem cell (CSC) properties with self-renewal, multipotency, and a unique potential for tumor initiation. HA has been shown to stimulate a variety of CSC functions including self-renewal, clone formation and differentiation. This review article will present current evidence for the existence of a unique small population of CD44v3^high^ALDH^high^-expressing CSCs in HNSCC. A special focus will be placed on the role of HA/CD44-induced oncogenic signaling and histone methyltransferase, DOT1L activities in regulating histone modifications (via epigenetic changes) and miRNA activation. Many of these events are essential for the CSC properties such as Nanog/Oct4/Sox2 expression, spheroid/clone formation, self-renewal, tumor cell migration/invasion, survival and chemotherapeutic drug resistance in HA-activated head and neck cancer. These newly-discovered HA/CD44-mediated oncogenic signaling pathways delineate unique tumor dynamics with implications for defining the drivers of HNSCC progression processes. Most importantly, the important knowledge obtained from HA/CD44-regulated CSC signaling and functional activation could provide new information regarding the design of novel drug targets to overcome current therapeutic drug resistance which will have significant treatment implications for head and neck cancer patients.

## 1. Hyaluronan (HA) Metabolism in Head and Neck Cancer Progression

Head and Neck Squamous cell carcinoma (HNSCC) is an aggressive malignant neoplasm associated with major morbidity and mortality. The three-year survival rate for patients with advanced-stage HNSCC who are treated with standard therapy is only 30% to 50% [1,2,3,4]. Resistance to standard therapy continues to be a limiting factor in the treatment of HNSCC. Nearly 40% to 60% of patients with HNSCC subsequently develop locoregional recurrences or distant metastases [1,2,3,4]. Presently, very little information is available regarding the cellular and molecular mechanisms involved in regulating HNSCC cell signaling and chemotherapeutic responses.

**Figure 1:**
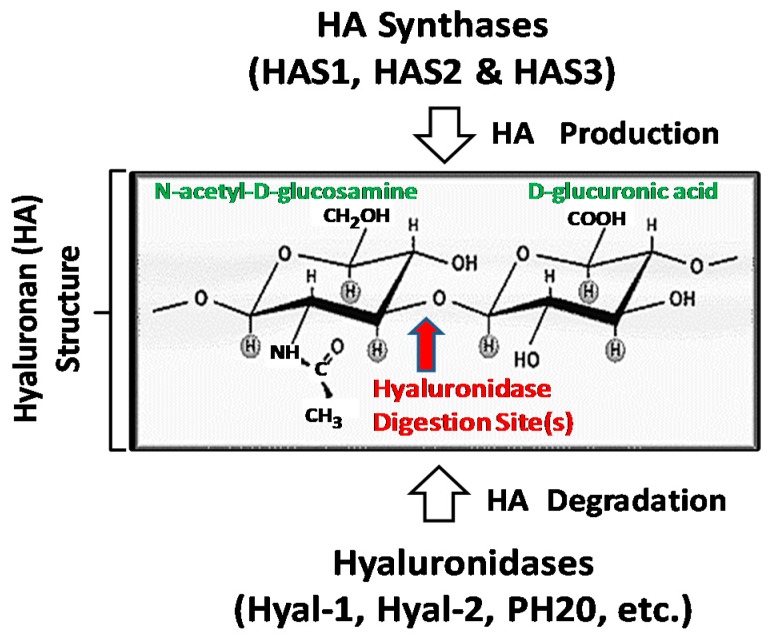
Hyaluronan (HA) structure regulated by HA synthases and hyaluronidases.

Hyaluronan (HA), one of the major extracellular matrix (ECM) components, is known to contain repeating disaccharides units, d-glucuronic acid and *N*-acetyl-d-glucosamine (Figure 1) [5,6]. It is often accumulated at tumor cell–matrix attachment sites and is closely associated with tumorigenesis and metastasis [5,6]. HA synthesis or production is regulated by at least three mammalian HA synthase isozymes including HAS1 (HA synthase 1), HAS2 (HA synthase 2) and HAS3 (HA synthase 3) [7,8,9,10]. All three HAS genes are located on different chromosomes. Structurally, all three HAS isozymes appear to share a great deal of sequence homology [11,12,13]. Both HAS1 and HAS2 synthesize large-size of HA (~1 × 10^6^–1 × 10^7^ Da) [11,12] which are known to be important for cell proliferation, angiogenesis and development [13]. HAS3 synthesizes small-size HA (~1 × 10^5^ Da) [11,12] which appears to contribute to many malignant behaviors of many different cell types [11,12]. HAS2 has been shown to express in head and neck cell lines [14]. Downregulation of HAS2 expression resulted in an inhibition of tumor cell growth and migration as well as an enhancement of cisplatin sensitivity, suggesting the importance of tumor cell HA production to promote in vitro tumor progression behaviors in oral cancer cells [14]. Overexpression of HAS2 in oral cavity carcinoma clinical specimens was also correlated with poor clinicopathologic characteristics and worse disease-free survival [14]. These observations strongly support the contention that HAS expression is closely associated with HNSCC progression. Thus, differential HAS expression/activities and unique HA-size production could selectively influence oncogenic signaling pathways and malignant properties leading to tumor progression.

Large-size HA polymers can be digested or degraded into smaller-sized HA fragments by acid-active hyaluronidases [15] (Figure 1). Overexpression of hyaluronidases has been associated with solid tumor metastases [16]. Presently, at least six hyaluronidase-like gene sequence products (e.g., Hyal-1, Hyal-2, Hyal-3, Hyal-4, *PHYAL-1*, and PH20 (or Spam1)) have been reported [17]. For example, the genes of Hyal-1 and Hyal-2 (the major hyaluronidases in human somatic tissues) are detected as tight clusters on chromosome 3p21.3. Hyal-1 is enriched in head and neck cancer [18]. Hyal-2 initially cleaves high-molecular-weight (HMW) large-size HA into ~20 kDa fragments, which are further digested into smaller size fragments by Hyal-1 [19,20]. Interestingly, low-molecular-weight (LMW) HA or small HA fragments, rather than high-molecular-weight (HMW) large HA, have been suggested to be essential for cancer progression in terms of invasion and metastasis [21]. Hyal-3 expression is detected in fibroblasts when they are undergone chondrocyte differentiation [17,22]. Hyal-4 could be a chondroitinase and *PHYAL*-1, a pseudogene appears to be untranslated in humans [23]. PH20 (or Spam1) belongs to a neutral-active hyaluronidase and is detected primarily in testes and in a variety of cancers including HNSCC tumors [18,24,25]. Most importantly, the unique HA-enriched microenvironment appears to serve as a stem cell niche and participates in cellular signaling and a variety of normal and cancer stem cell CSC)-specific functions [26,27].

## 2. CD44 (a HA Receptor) in Cancer Stem Cells (CSCs) and Tumor Progression

**Figure 2:**
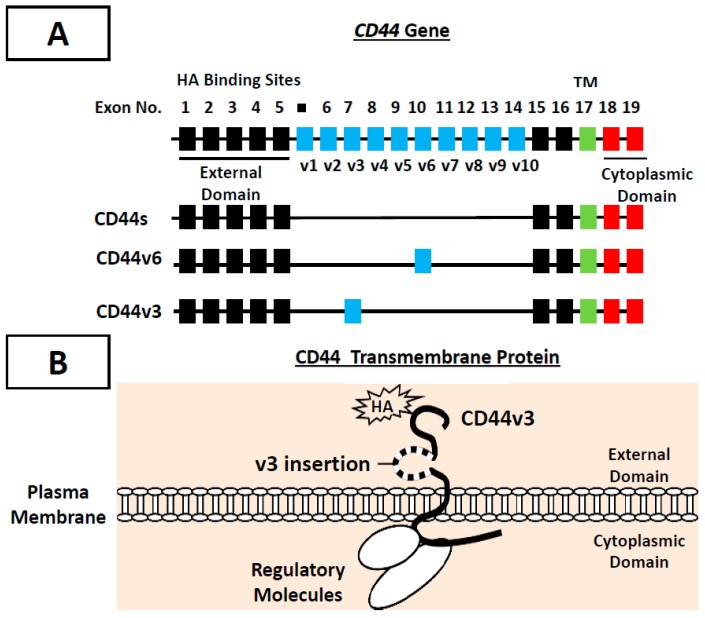
Illustration of CD44 gene and alternative spliced variants (e.g., CD44v3 and CD44v6 isoforms) which contain several structural domains including external domain, transmembrane (TM) and intracellular domain (**A**); and demonstration of CD44v3 protein which displays the HA binding domain at the external N-terminal region and the signaling regulator binding sites at the cytoplasmic domain (**B**).

CD44, a family of transmembrane glycoproteins is expressed in a variety of cells and tissues including head and neck cells and HNSCC tissues [28,29,30,31]. The presence of high levels of CD44 variant (CD44v) isoforms is emerging as an important metastatic tumor marker in many cancers including head and neck cancer [28,29,30,31]. *CD44* gene is known to undergo alternative splicing mechanisms and produces a variety of CD44 isoforms including CD44s, CD44v3, CD44v6, etc. [32,33] (Figure 2A). Mack and Gires showed that both CD44s and CD44v6 can be detected in 60–95% and 50–80%, respectively, of normal epithelia, whereas, in moderately differentiated carcinoma, the level of CD44s and CD44v6 only increase slightly [34]. Since CD44s and CD44v6 fail to distinguish normal from benign or malignant epithelia, these two CD44 isoforms cannot be used as reliable tumor markers for monitoring HNSCC progression. In contrast, CD44v3 can be detected in lymph metastasis and is closely involved in promoting metastasis and head and neck cancer progression [28,29,30,31]. The v3 exon (but not other variants) product of the CD44 molecule is unique, being the only exon that has a heparin sulfate (HS) assembly site (33). These v3 exon-containing variants carrying HS side chains bind and present heparin-binding growth factors and cytokines, such as FGF and heparin-binding epidermal growth factor (EGF), which may generate various functional consequences. These findings suggest that different CD44 isoforms may participate in distinct biological activities.

It has been well documented that all CD44 isoforms contain HA-binding domain located at the N-terminal region of the extracellular domain [35] (Figure 2A). The binding of CD44 isoforms to hyaluronan affects cell adhesion to extracellular matrix components and is implicated in the stimulation of aggregation, proliferation, migration, and angiogenesis [28,29,30,31,36]. The intracellular domain of CD44 isoforms (e.g., CD44v3) selectively interacts with cytoplasmic regulators (e.g., oncogenic molecules and cytoskeletal proteins) and effectively transmits cellular signaling [28,29,30,31,37,38,39,40]. Therefore, CD44 isoforms likely provide a close linkage between matrix components (HA) and cytoplasmic regulators (Figure 2B). Recent studies have shown that CD44 is also detected in tumor stem cells which have the unique ability to initiate tumor cell-specific properties [41]. In fact, CD44 is proposed to be one of the important surface markers for cancer stem cells [41]. Both CD44v isoforms and HA are overexpressed at sites of tumor attachment [42].

Accumulating evidence indicates that most tumors contain special subpopulations of cells, which appear to be highly tumorigenic and are involved in tumor progression. These “Cancer Stem Cells (CSCs)” or “Tumor-Initiating Cells (TICs)” share several common properties of normal stem cells [43,44]. However, some CSCs have altered their ability to regulate normal stem cell numbers, pluripotency, and lineage-dependent differentiation [43,44]. CSCs frequently undergo cell division/growth and/or clone formation and differentiation to expand the stem cell population [43,44]. They are also capable of generating self-renewal, maintaining quiescence, and displaying multipotentiality [43,44]. Consequently, CSC-derived tumor cells often display a variety of properties which are different from the parental tumor cells. These studies suggest that tumor formation could be a result of a variety of CSCS signaling modifications and/or functional alterations. Furthermore, CSCs have been shown to be chemoresistant and are possibly responsible for cancer relapse.

**Figure 3:**
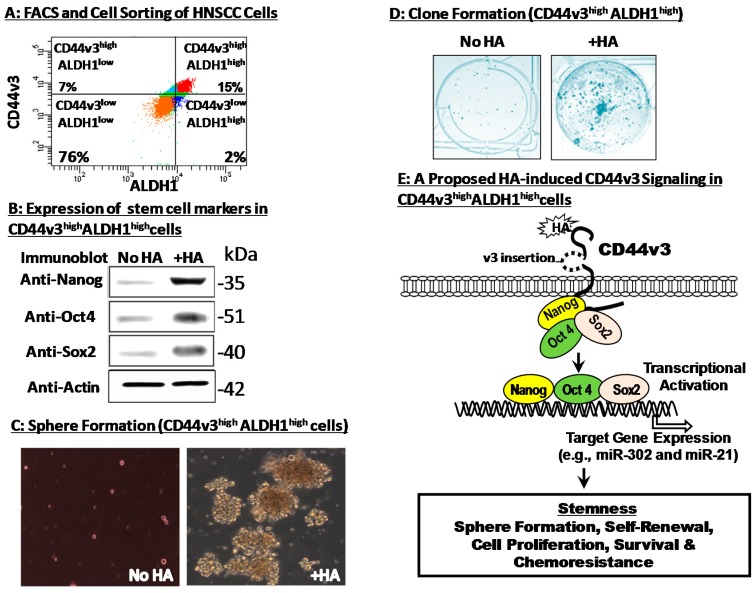
Characterization of cancer stem cell-like population from tumor-derived HNSCC (HSC-3 cells). (**A**) Flow cytometry analyses of HSC-3 tumor cell populations: Specifically, HSC-3 cells were stained with both allophycocyanin (APC)-labeled anti-CD44v3 antibody (recognizing the v3-specific domain of CD44) and ALDEFLUOR (measuring an ALDH1 enzymatic activity) followed by processing through multicolor fluorescence-activated cell sorter (FACS) and cell sorting. (Top right quad represents CD44v3^high^ALDH1^high^ cells (15%); Top left quad represents CD44v3^high^ALDH1^low^ cells (7%); Bottom right quad represents CD44v3^low^ALDH1^high^ cells (2%); and Bottom left quad represents CD44v3^low^ALDH1^low^ cells (76%). A FACS-fluorescence-activated cell sorter was then used to isolate CD44v3^high^ALDH1^high^ cells or CD44v3^low^ALDH1^low^ cells for the study; (**B**) detection of stem cell markers Nanog, Oct4 and Sox2 in CD44v3^high^ALDH1^high^ cells (treated with no HA or with HA) using immunoblot analyses. (**C**) Sphere formation of CD44v3^high^ALDH1^high^ cells in: the absence of HA; or the presence of HA. (**D**) Clone formation of CD44v3^high^ALDH1^high^ cells in: the absence of HA; or the presence of HA. (**E**) A proposed HA-induced CD44v3 signaling in CD44v3^high^ALDH1^high^ cells. The binding of HA to CD44v3-expressing CSCs promotes Nanog/Oct4/Sox2 association with CD44v3 first followed by Nanog/Oct4/Sox2 translocation to the promoter sites of target genes. Subsequently, Nanog/Oct4/Sox2-specific target genes (e.g., miR-302 and miR-21) are expressed leading to stemness properties (e.g., sphere formation, self-renewal, cell proliferation, survival and chenoresistance) and tumor progression.

Head and neck cancer (HNSCC) tumors contain a subpopulation of CD44s^high^-expressing CSCs which can generate phenotypically distinct cells [45]. When these CSCs were injected into immuno-deficient mice, the formation of heterogeneous tumors was observed [45]. These observations suggest that CD44s^high^-expressing CSCs may exhibit stem cell-like properties (e.g., self-renewal, clone formation and differentiation) which are responsible for the generation of heterogeneous tumor cell populations. In fact, CD44s has been widely used as one of the most important stem cell surface markers in several studies [41,45]. Aldehyde dehydrogenase-1 (ALDH1), a detoxifying enzyme for intracellular aldehyde oxidation, has also been considered as a stem cell marker in both normal tissues and human HNSCC [46,47]. Recently, several studies indicate that overexpression of both CD44 variant isoform (CD44v3) and ALDH1 correlates well with tumor formation and HNSCC progression [28,29,30,31,46,47]. Specifically, a subpopulation characterized by overexpression of both CD44v3 and ALDH1 (so-called CD44v3^high^ALDH1^high^ cells) was isolated using anti-CD44v3 antibody plus ALDH1-specific reagent and FACS-fluorescence-activated cell sorter [48,49,50] (Figure 3A). Injection of CD44v3^high^ALDH1^high^-expressing cells into NOD/SCID mice generated phenotypically distinct cells in heterogeneous tumors [48,49,50]. Most importantly, a high efficiency of tumor formation was detected by injecting as few as ~50 CD44v3^high^ALDH1^high^ cells (but not CD44v3^low^ALDH1^1ow^ cells or unsorted cells) into NOD/SCID mice [48,49,50]. These findings suggest that the high level of tumorigenic effect induced by CD44v3^high^ALDH1^high^ cells (but not CD44v3^low^ALDH1^1ow^ cells or unsorted cells) may be caused by unique tumor initiation properties of these subpopulations of HNSCC cells [48].

## 3. Hyaluronan–CD44 Interaction Stimulates Stem Cell Marker Expression, Stemness Properties and Chemoresistance in CD44v3^high^ALDH1^high^ Head and Neck Cancer Stem Cells (CSCs)

One of the common properties of CD44v3^high^ALDH1^high^ CSCs isolated from HNSCC is the upregulation of cancer stem cell markers (e.g., Nanog, Sox2 and Oct4) which are known to function as transcriptional factors [48,49,50]. These three regulatory factors often form a combinational complex to stimulate specific genes targeting for the expression of stem cell properties including proliferation, self-renewal, and differentiation [51]. Overexpression of Nanog/Sox2/Oct4 has also been closely correlated with higher histological grades and poorer clinical survival in head and neck cancer [48,52,53,54,55,56]. Nanog is an important transcription factor that renders the reprogramming adult stems into germ-line-competent induced pluripotent stem (iPS) cells [57]. It is expressed not only in germ cell tumors, but also in many other tumors including HNSCC cells and tissues [48,53]. In HNSCC, high expression of Nanog has been shown to be associated with advanced cancer stages and HNSCC progression [48,53,55,56]. Sox2 (Sex determining region Y-box (2)) is known to be involved in the maintenance of embryonic stem cell pluripotency and in multiple developmental processes [53,58,59,60]. Due to the weak DNA binding ability, Sox2 often forms complexes with other transcription factors [58]. Increasing numbers of studies regarding the association between SOX2 and malignant tumors (e.g., head and neck cancer) have been reported [59,60]. Overexpression of SOX2 is associated with lymph node metastasis in oral cancer [54]. Another stem cell marker, Oct4 is known to act as an important transcriptional factor which binds to an octameric sequence motif containing the AGTCAAAT consensus sequence to cause both the onset and maintenance of pluripotency during embryonic development [61]. Oct4 also interacts with other stem cell markers, such as Nanog and Sox2, by forming a large regulatory complexes or networks that stimulate pluripotency and/or block differentiation [58,62]. Moreover, stem cell profiling in head and neck cancer reveals an Oct-4 expressing subpopulation with properties of chemoresistance [63]. Most importantly, the expression of stem cell markers such as Nanog, Sox2 and Oct4 is closely associated with HNSCC patient’s prognosis, pathological stages, cancer recurrence and therapy resistance [55,56]. Therefore, searching for new cancer stem cell targets or designing drugs to manipulate the known molecular targets in CSCs could be useful for improvements in clinical outcomes of the disease.

It has been reported that self-renewal, proliferation and differentiation regulated by Nanog/Sox2/Oct4 are the hallmark of stem cell properties that allow CSCs to produce both new cancer stem cells and phenotypically diverse cancer cells with different proliferative potential [43,44]. All three stem cell markers (i.e., Nanog, Oct4 and Sox2) have been shown to be upregulated (Figure 3B) and can be recruited into the CD44v3-associated membranes during HA-mediated signaling in these head and neck CSCs [48,49] (Figure 3E). Recent studies also indicate that HA binding to CD44 induces upregulation of Nanog, Sox2 and Oct4 expression and activates a variety of important target genes such as miR-302 and miR-21 in CD44v3^high^ALDH1^high^ head and neck CSCs and breast tumor cells, respectively [48,64,65] (Figure 3E). Most importantly, HA-mediated CD44 signaling promotes spheroid (Figure 3C) and clone formation (Figure 3D), as well as cell growth/self-renewal properties of CD44v3^high^ALDH1^high^ head and neck CSCs [48,49]. These findings strongly suggest that HA–CD44 interaction is tightly linked to the onset of stem cell marker (Nanog/Sox2/Oct4) expression and the subsequent stem cell functions in CSCs isolated from HNSCC.

Head and neck cancer treatment commonly involves the use of cisplatin-related chemotherapeutic drugs. HNSCC tumor resistance to chemotherapy is often caused by the presence of anti-apoptotic regulators [31,66]. IAP family (e.g., cIAP-1, cIAP-2 and XIAP) appears to play an important role in suppressing apoptosis and promoting oncogenesis [66]. IAPs not only block the activities of several caspases and pro-caspases such as caspase-3 and caspase-7 [67], but also prevent anti-cancer drug-induced tumor cell death [67]. Even though some attention has been paid to the involvement of other anti-apoptotic proteins (e.g., Bcl-xL) in anti-apoptosis and chemoresistance of breast tumor cells in the presence of HA [68], recently, there is a significant interest of investigating a possible role of IAPs in HA-treated HNSCC tumor resistance [69]. Recent results show that there is a significant elevation of IAP protein (e.g., cIAP-1, cIAP-2 and XIAP) expression in CD44v3^high^ALDH1^high^ head and neck CSCs following HA treatment [48,49,50]. Upon treatment of cisplatin, these CSCs show a reduction in apoptosis and cell death (possibly due to an increase of IAP production and tumor cell survival) and a significant increase in cisplatin-resistance [48,49,50] (Figure 3E). Taken together, these findings strongly suggest that HA–CD44 interaction plays an important role in promoting Nanog/Oct4/Sox2 upregulation, stemness properties, tumorigenesis and chemoresistance in CD44v3^high^ALDH1^high^ head and neck CSCs, which are important contributors to HNSCC progression. Another cell surface marker is the single-chain sialoglycoprotein CD24, which is also associated with cancer stem cell characteristics in colorectal and pancreatic cancer, while head and neck cancer cells and breast cancer cells with CD44^+^CD24^−/low^ expression are highly tumorigenic [70,71,72]. However, the question of whether HA displays any effect on CD44^+^CD24^−/low^ cancer stem cells is presently unknown. 

## 4. Hyaluronan (HA) Promote miRNA-10b Expression, Tumor Cell Invasion and Chemoresistance in Head and Neck Cancer Stem Cells (CSCs)

It has been well-documented that cytoskeleton functions are directly involved in tumor cell invasion of surrounding tissue, and metastasis [73]. During the search for cellular regulators that are involved in controlling cytoskeleton function and cell migration/invasion in HNSCC, certain microRNAs (miRNAs) (~22 nucleotides) known to function as RNA interference for gene regulation [74] were identified. Alterations of miRNAs are often associated with the pathogenesis of HNSCC [75]. Several previous studies demonstrated that certain oncogenic microRNAs promote the cell functions of CD44v3^high^ALDH1^high^ (CSC-like) subpopulation isolated from HNSCC (HSC-3) [48,49,50]. However, the information regarding the regulation of miRNA production by matrix component(s) in head and neck cancer CSCs is very limited.

One general concept that has emerged from recent studies is that HA fragments (small vs. mid-size HAs) and their larger precursor molecules (i.e., intact HA) may be involved in regulating distinct biological activities including miRNAs [48,49,50,76,77]. For example, the generation of different sizes of HA fragments from large/intact HA in the ECM occurs during periods of cell growth, migration, differentiation and development as well as injury-related repairs [9,10]. Several studies indicate that there is a selective activation of transcriptional activation and differentiation by large HA fragments, but not small HA fragments. Other cellular functions such as cell proliferation and migration appear to be preferentially regulated by small-size HA fragments, but not large-size HA. [49,76,77]. Recently, a panel of stem cell-related miRNAs including miR-10b, miR-27b, miR-373, miR-181, miR-34a and miRNA-145 has been shown to be preferentially up-regulated by 200 kDa-HA in head and neck CSCs [49]. In contrast, the other sizes of HA (e.g., 5 kDa-HA, 20 kDa-HA and 700 kDa-HA) fail to induce stem cell-specific miRNA production in the CD44v3^high^ALDH1^high^ (CSC-like) subpopulation from HNSCC cells [49]. Most noticeably, miR-10 has been shown to undergo the highest level of stimulation by 200 kDa-HA in CSCs isolated from HNSCC [49]. In a previous study, miRNA-10b was found to be overexpressed in malignant glioma in addition to the overexpression of RhoC and uPAR which are contributors to glioma invasion and migration [78]. Most importantly, 200 kDa-HA/CD44-activated miR-10b production also plays an important role in upregulating the cytoskeleton regulator, RhoC and CSC migration/invasion [49,50]. Treatment of CSCs with an anti-miR-10 inhibitor significantly decreases RhoC expression and blocks CSC migration/invasion [49,50]. These findings suggest that miR-10 is closely involved in regulating cytoskeleton function required for CSC migration/invasion and the miR-10b inhibitor may be considered as a promising new anti-cancer agent.

Treatment of head and neck cancer CSCs with 200 kDa-HA also causes cisplatin resistance [49]. Survival proteins such as Inhibitors of Apoptosis Proteins (IAPs) are frequently upregulated in CSCs following HA treatment [48,49,50]. Importantly, high levels of IAPs in CSCs increase cell survival due to the binding of IAPs to caspases and the suppression of apoptosis [48,49,50]. A recent study indicates that the expression of IAPs such as c-IAP-1 significantly increased in CD44v3^high^ALDH1^high^ head and neck cancer CSCs treated with 200 kDa-HA [49]. There is only a relatively low level of c-IAP-1 expression detected with other sizes of HAs (e.g., 5 kDa-HA, 20 kDa-HA or no HA treatment) [49]. These findings clearly indicate that the survival proteins such as c-IAP-1 is up-regulated in the CSC subpopulation isolated from HNSCC cells following 200 kDa-HA treatment. Further analyses show that the addition of 200 kDa-HA in CSCs not only significantly increases miR-10b expression, but also greatly decreases the ability of cisplatin to induce tumor cell death [49]. These observations strongly suggest that 200 kDa-HA causes both a decrease in tumor cell death and an increase in tumor cell survival leading to the enhancement of chemoresistance [49]. Moreover, downregulation of miR-10b using anti-miR-10b inhibitor significantly reduces the survival and increases cisplatin sensitivity in 200 kDa-treated CSCs [49]. These findings clearly suggest that downregulation of the 200 kDa-HA-induced miR-10 b function (by anti-miR-10 b treatment) may represent a new target for therapeutic agents designed to cause head and neck cancer CSCs to undergo cell death and remain chemotherapy sensitive.

## 5. HA–CD44 Interaction Promotes Histone Methyltransferase, DOT1L Expression and Epigenetic Modification in Head and Neck Cancer Stem Cells

Epigenetic changes such as histone methylation have emerged as one of the important regulatory processes in the chromatin structural modifications and the gene expression alterations during cancer progression [79]. Methylation of histone H3 at lysine 79 (H3K79) is highly conserved among most eukaryotic species. In budding yeast, nearly 90% of histone H3 displays either monomethylation (H3K79me1), dimethylation (H3K79me2) or trimethylation (H3K79me3) at lysine 79, all catalyzed exclusively by the histone methyltransferase, DOT1 [80,81]. In *Saccharomyces cerevisiae*, DOT1 was initially identified as a disruptor of telomeric silencing. The orthologs of DOT1 appear to be evolutionarily conserved from yeast to mammals [80,81]. Both DOT1 and the mammalian DOT1L (DOT1-like protein) function as H3K79 methyltransferases in the regulation of histone H3K79 methylation and transcriptional activation [82]. Specifically, DOT1/DOT1L-mediated H3K79 methylation is known to be involved in the control of transcriptional activity required for cell cycle, meiotic checkpoint and the DNA damage checkpoint [83]. An earlier study indicated that mammalian DOT1L participates in proliferation and differentiation in embryonic stem (ES) cells [84]. It has also been reported that aberrant H3K79 methylation by DOT1L occurs in Mixed Lineage Leukemia (MLL) [85]. Treatment of *MLL*-rearranged leukemia with EPZ00477 or EPZ-5676 (potent and selective amino nucleoside inhibitors of DOT1L histone methyltransferase activity) causes cell death in acute leukemia lines bearing *MLL* translocations [86,87]. Furthermore, downregulation of H3K79 methyltransferase, DOT1L, using specific DOT1LsiRNA also reduced proliferation of lung cancer cells [88]. These findings indicate that H3K79 methyltransferase, DOT1L, plays an important role in promoting cancer formation.

**Figure 4:**
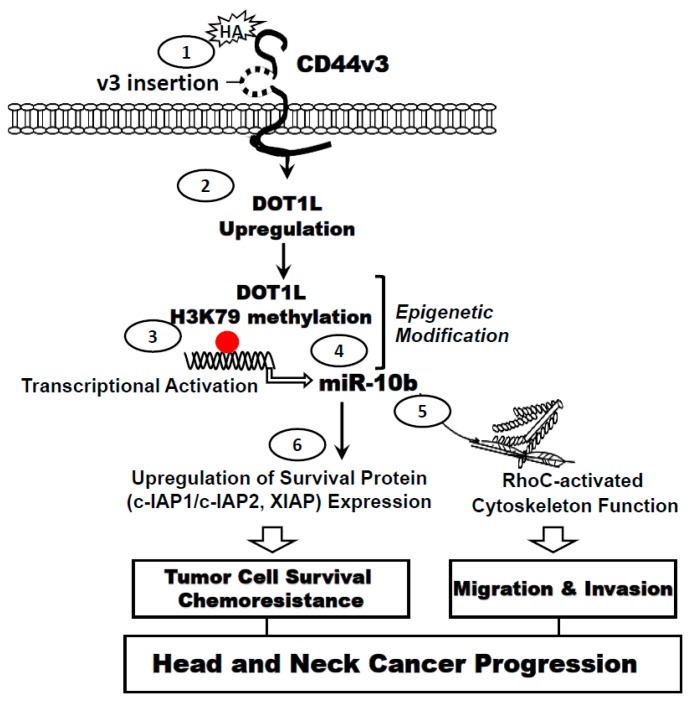
A summary for DOT1L-H3K79 methylation signaling in influencing miRNA-10 gene expression and production as well as Cancer Stem Cell (CSC) functions in HA-treated CD44v3^high^ALDH1^high^ cells. The binding of HA (**Step 1**) to CD44v3-expressing CSCs enhances DOT1L upregulation (**Step 2**) and DOT1L/H3K79 methylation-mediated epigenetic changes (**Step 2**), resulting in methyl-H3K79 binding to miR-10b promoter (**Step 3**), and miR-10b gene expression/production (**Step 4**). The expressed miR-10b then promotes upregulation of RhoC (cytoskeleton activator) and CSC-mediated invasion (**Step 5**). Moreover, miR-10b also induces survival protein, IAP (cIAP-1/cIAP-2 and XIAP) expression, HNSCC cell anti-apoptosis/survival and chemoresistance (**Step 6**). Red dot represents DOT1L/H3K79-mediated histone modifications (via epigenetic changes). Taken together, these findings suggest that targeting HA-mediated DOT1L/H3K79 methylation pathways and miR-10b function may provide a new drug target to sensitize cancer stem cell apoptosis/death and overcome cisplatin resistance in CSC-derived HNSCC cells.

A recent study indicates that histone methyltransferase, DOT1L can be upregulated by HA in CD44v3^high^ALDH1^high^ head and neck CSCs [50]. Previously, tumor invasion and migration-associated miR-10b has been shown to contain several predicted E-box elements at its upstream promoter region [89]. DOT1L-activated H3K79 methylation appears to be directly involved in the binding of the E-box elements at the miR-10b promoter leading to miR-10b gene expression and production in head and neck cancer CSCs treated with HA [50]. Further analyses indicate that the stimulation of microRNA-10b (miR-10b) expression is DOT1L-specific and HA/CD44-dependent in CD44v3^high^ALDH1^high^ CSCs. This process subsequently results in the overexpression of RhoGTPases and survival proteins leading to tumor cell invasion and cisplatin resistance. These findings suggest that HA/CD44-activated miR-10 production plays an important role in upregulating the cytoskeleton regulator, RhoC in CSC-like cells. Most importantly, we found that downregulation of HA-activated DOT1L signaling or miR-10b production by treating CSCs with DOT1L siRNA or anti-miR-10b inhibitor, respectively, not only reduces RhoGTPase (RhoC) expression and impairs tumor cell migration/invasion but also enhances chemosensitivity [50]. These observations clearly establish a causal link between DOT1L/H3K79 methylation and miR-10b function including cytoskeleton-regulated CSC invasion and chemoresistance in HA-treated CSCs. Taken together, these findings strongly support the contention that histone methyltransferase, DOT1L-associated epigenetic changes induced by HA play pivotal roles in miR-10 production leading to upregulation of RhoGTPase and survival proteins. These events are critically important for the acquisition of cancer stem cell properties including tumor cell invasion and chemotherapy resistance in HA/CD44-activated CD44v3^high^ALDH1^high^ head and neck cancer CSCs (Figure 4).

## 6. Conclusions

This review has placed a special focus on the properties of a unique subpopulation of HNSCC cells (i.e., CD44v3^high^ALDH1^high^), which displays CSC characteristics with high tumorigenic potential and chemoresistance. These CD44v3^high^ALDH1^high^-expressing CSCs appear to be closely involved in the pathobiology of head and neck squamous cell carcinomas. Most importantly, matrix hyaluronan (HA) interaction with CD44v3 plays a pivotal role in regulating stem cell marker expression, stemness properties (e.g., spheroid formation, clone formation, self-renewal, differentiation) and chemoresistance in these head and neck cancer stem cells. Moreover, histone methyltransferase, DOT1L/H3K79 methylation-associated epigenetic changes induced by matrix HA has been shown to directly regulate miR-10 production leading to up-regulation of RhoGTPase and survival proteins as well as migration/invasion and chemoresistance in CD44v3^high^ALDH1^high^-expressing CSCs. In the future, cancer stem cell-based signaling perturbation therapies may be developed to downregulate stem cell transcription factors and/or histone methyltransferase, DOT1L (using Nanog/Oct4/Sox2/DOT1L-specific siRNA and shRNA vector approaches or DOT1L inhibitors) and/or to silence miR-10b (using anti-miR-10b inhibitor) for blocking HA/CD44v3-mediated Nanog/Oct4/Sox2 signaling and DOT1L/miR-10b expression/function as well as chemoresistance and HNSCC progression. Taken together, these recently discovered matrix hyaluronan (HA)/CD44v3-mediated signaling pathways and DOT1L-specific epigenetic regulation in head and neck CSCs could provide unique molecular targets and specific therapeutic drugs for the treatment of head and neck cancer.

## References

[1] Parkin D.M., Bray F., Ferlay J., Pisani P. (2002). Global cancer statistics, 2002. CA Cancer J. Clin..

[2] Haddad R.I., Shin D.M. (2008). Recent advances in head and neck cancer. N. Engl. J. Med..

[3] Leemans C.R., Braakhuis B.J., Brakenhoff R.H. (2011). The molecular biology of head and neck cancer. Nat. Rev. Cancer.

[4] Pfister D.G., Ang K.K., Brizel D.M., Burtness B.A., Cmelak A.J., Colevas A.D., Dunphy F., Eisele D.W., Gilbert J., Gillison M.L. (2011). Head and neck cancers. J. Natl. Compr. Cancer Netw..

[5] Toole B.P., Hay E.D. (1991). Proteoglycans and hyaluronan in morphogenesis and differentiation. Cell Biology of Extracellular Matrix.

[6] Chanmee T., Ontong P., Itano N. (2016). Hyaluronan: A modulator of the tumor microenvironment. Cancer Lett..

[7] Weigel P.H., Hascall V.C., Tammi M. (1997). Hyaluronan synthases. J. Biol. Chem..

[8] Itano N., Kimata K. (1996). Expression cloning and molecular characterization of HAS protein, a eukaryotic hyaluronan synthase. J. Biol. Chem..

[9] Itano N., Kimata K. (1998). Hyaluronan synthase: New directions for hyaluronan research. Trends Glycosci. Glycotechnol..

[10] Weigel P.H., Baggenstoss B.A., Washburn J.L. (2017). Hyaluronan synthase assembles hyaluronan on a [GlcNAc(β1,4)]n-GlcNAc(α1→)UDP primer and hyaluronan retains this residual chitin oligomer as a cap at the nonreducing end. Glycobiology.

[11] Spicer A.P., McDonald J.A. (1998). Characterization and molecular evolution of a vertebrate hyaluronan synthase gene family. J. Biol. Chem..

[12] Shyjan A.M., Heldin P., Butcher E.C., Yoshino T., Briskin M.J. (1996). Functional cloning of the cDNA for a human hyaluronan synthase. J. Biol. Chem..

[13] Tammi R.H., Passi A.G., Rilla K., Karousou E., Vigetti D., Makkonen K., Tammi M.I. (2011). Transcriptional and post-translational regulation of hyaluronan synthesis. FEBS J..

[14] Wang S.J., Earle C., Wong G., Bourguignon L.Y. (2013). Role of hyaluronan synthase 2 to promote CD44-dependent oral cavity squamous cell carcinoma progression. Head Neck.

[15] Stern R., Jedrzejas M.J. (2006). Hyaluronidases: Their genomics, structures, and mechanisms of action. Chem. Rev..

[16] Nykopp T.K., Rilla K., Tammi M.I., Tammi R.H., Sironen R., Hämäläinen K., Kosma V.M., Heinonen S., Anttila M. (2010). Hyaluronan synthases (HAS1-3) and hyaluronidases (HYAL1-2) in the accumulation of hyaluronan in endometrioid endometrial carcinoma. BMC Cancer.

[17] Csoka A.B., Frost G.I., Ster N.R. (2001). The six hyaluronidase-like genes in the human and mouse genomes. Matrix Biol..

[18] Franzmann E.J., Schroeder G.L., Goodwin W.J., Weed D.T., Fisher P., Lokeshwar V.B. (2003). Expression of tumor markers hyaluronic acid and hyaluronidase (HYAL1) in head and neck tumors. Int. J. Cancer.

[19] Lepperdinger G., Müllegger J., Kreil G. (2001). Hyal2-less active, but more versatile?. Matrix Biol..

[20] Harada H., Takahashi M. (2007). CD44-dependent intracellular and extracellular catabolism of hyaluronic acid by hyaluronidase-1 and -2. J. Biol. Chem..

[21] Wu M., Cao M., He Y., Liu Y., Yang C., Du Y., Wang W., Gao F. (2015). A novel role of low molecular weight hyaluronan in breast cancer metastasis. FASEB J..

[22] Flannery C.R., Little C.B., Hughes C.E., Caterson B. (1998). Expression and activity of articular cartilage hyaluronidases. Biochem. Biophys. Res. Commun..

[23] Csoka A.B., Scherer S.W., Stern R. (1999). Expression analysis of six paralogous human hyaluronidase genes clustered on chromosomes 3p21 and 7q31. Genomics.

[24] Cherr G.N., Yudin A.I., Overstreet J.W. (2001). The dual functions of GPI-anchored PH-20: Hyaluronidase and intracellular signaling. Matrix Biol..

[25] Beech D.J., Madan A.K., Deng N. (2002). Expression of PH-20 in normal and neoplastic breast tissue. J. Surg. Res..

[26] Haylock D.N., Nilsson S.K. (2006). The role of hyaluronic acid in hemopoietic stem cell biology. Regen. Med..

[27] Astachov L., Vago R., Aviv M., Nevo Z. (2011). Hyaluronan and mesenchymal stem cells: From germ layer to cartilage and bone. Front. Biosci..

[28] Franzmann E.J., Weed D.T., Civantos F.J., Goodwin W.J., Bourguignon L.Y. (2001). A novel CD44v3 isoform is involved in head and neck squamous cell carcinoma progression. Otolaryngol. Head Neck Surg..

[29] Wang S.J., Wreesmann V.B., Bourguignon L.Y. (2007). Association of CD44v3-containing isoforms with tumor cell growth, migration, matrix metalloproteinase expression, and lymph node metastasis in head and neck cancer. Head Neck.

[30] Wang S.J., Wong G., de Heer A.M., Xia W., Bourguignon L.Y. (2009). CD44 variant isoforms in head and neck squamous cell carcinoma progression. Laryngoscope.

[31] Wang S.J., Bourguignon L.Y. (2011). Role of hyaluronan-mediated CD44 signaling in head and neck squamous cell carcinoma progression and chemoresistance. Am. J. Pathol..

[32] Screaton G.R., Bell M.V., Jackson D.G., Cornelis F.B., Gerth U., Bell J.I. (1992). Genomic structure of DNA coding the lymphocyte homing receptor CD44 reveals 12 alternatively spliced exons. Proc. Natl. Acad. Sci. USA.

[33] Bennett K.L., Jackson D.G., Simon J.C., Tanczos E., Peach R., Modrell B., Stamenkovic I., Plowman G., Aruffo A. (1995). CD44 isoforms containing exon V3 are responsible for the presentation of heparin-binding growth factor. J. Cell Biol..

[34] Mack B., Gires O. (2008). CD44s and CD44v6 expression in head and neck epithelia. PLoS ONE.

[35] Peach R.J., Hollenbaugh D., Stamenkovic I., Aruffo A. (1993). Identification of hyaluronic acid binding sites in the extracellular domain of CD44. J. Cell Biol..

[36] Lokeshwar V.B., Iida N., Bourguignon L.Y. (2002). The Cell adhesion molecule, GP116, is a new CD44 variant (ex14/v10) involved in hyaluronic acid binding and endothelial cell proliferation. J. Biol. Chem..

[37] Bourguignon L.Y., Lokeshwar V.B., Chen X., Kerrick W.G. (1993). Hyaluronic acid-induced lymphocyte signal transduction and HA receptor (GP85/CD44)-cytoskeleton interaction. J. Immunol..

[38] Bourguignon L.Y. (2008). Hyaluronan-mediated CD44 activation of RhoGTPase signaling and cytoskeleton function promotes tumor progression. Semin. Cancer Biol..

[39] Bourguignon L.Y., Wong G., Earle C., Krueger K., Spevak C.C. (2010). Hyaluronan-CD44 interaction promotes c-Src-mediated twist signaling, microRNA-10b expression, and RhoA/RhoC up-regulation, leading to Rho-kinase-associated cytoskeleton activation and breast tumor cell invasion. J. Biol. Chem..

[40] Bourguignon L.Y., Shiina M., Li J.J. (2014). Hyaluronan-CD44 interaction promotes oncogenic signaling, microRNA functions, chemoresistance, and radiation resistance in cancer stem cells leading to tumor progression. Adv. Cancer Res..

[41] Al-Hajj M., Wicha M.S., Benito-Hernandez A., Morrison S.J., Clarke M.F. (2003). Prospective identification of tumorigenic breast cancer cells. Proc. Natl. Acad. Sci. USA.

[42] Yeo T.K., Nagy J.A., Yeo K.T., Dvorak H.F., Toole B.P. (1996). Increased hyaluronan at sites of attachment to mesentery by CD44-positive mouse ovarian and breast tumor cells. Am. J. Pathol..

[43] Dalerba P., Cho R.W., Clarke M.F. (2007). Cancer stem cells: Models and concepts. Annu. Rev. Med..

[44] Schulenburg A., Ulrich-Pur H., Thurnher D., Erovic B., Florian S., Sperr W.R., Kalhs P., Marian B., Wrba F., Zielinski C.C. (2006). Neoplastic stem cells: A novel therapeutic target in clinical oncology. Cancer.

[45] Prince M.E., Sivanandan R., Kaczorowski A., Wolf G.T., Kaplan M.J., Dalerba P., Weissman I.L., Clarke M.F., Ailles L.E. (2007). Identification of a subpopulation of cells with cancer stem cell properties in head and neck squamous cell carcinoma. Proc. Nat. Acad. Sci. USA.

[46] Chen Y.C., Chen Y.W., Hsu H.S., Tseng L.M., Huang P.I., Lu K.H., Chen D.T., Tai L.K., Yung M.C., Chang S.C. (2009). Aldehyde dehydrogenase 1 is a putative marker for cancer stem cells in head and neck squamous cancer. Biochem. Biophys. Res. Commun..

[47] Clay M.R., Tabor M., Owen J.H., Carey T.E., Bradford C.R., Wolf G.T., Wicha M.S., Prince M.E. (2010). Single-marker identification of head and neck squamous cell carcinoma cancer stem cells with aldehyde dehydrogenase. Head Neck.

[48] Bourguignon L.Y., Wong G., Earle C., Chen L. (2012). Hyaluronan-CD44v3 interaction with Oct4-Sox2-Nanog promotes miR-302 expression leading to self-renewal, clonal formation, and cisplatin resistance in cancer stem cells from head and neck squamous cell carcinoma. J. Biol. Chem..

[49] Shiina M., Bourguignon L.Y. (2015). Selective activation of cancer stem cells by size-specific hyaluronan in head and neck cancer. Int. J. Cell Biol..

[50] Bourguignon L.Y., Wong G., Shiina M. (2016). Up-regulation of histone methyltransferase, DOT1L, by matrix hyaluronan promotes microRNA-10 expression leading to tumor cell invasion and chemoresistance in cancer stem cells from head and neck squamous cell carcinoma. J. Biol. Chem..

[51] Kashyap V., Rezende N.C., Scotland K.B., Shaffer S.M., Persson J.L., Gudas L.J., Mongan N.P. (2009). Regulation of stem cell pluripotency and differentiation involves a mutual regulatory circuit of the NANOG, OCT4, and SOX2 pluripotency transcription factors with polycomb repressive complexes and stem cell microRNAs. Stem Cells Dev..

[52] Wang Q., He W., Lu C., Wang Z., Wang J., Giercksky K.E., Nesland J.M., Suo Z. (2009). Oct3/4 and Sox2 are significantly associated with an unfavorable clinical outcome in human esophageal squamous cell carcinoma. Anticancer Res..

[53] Chiou S.H., Yu C.C., Huang C.Y., Lin S.C., Liu C.J., Tsai T.H., Chou S.H., Chien C.S., Ku H.H., Lo J.F. (2008). Positive correlations of Oct-4 and Nanog in oral cancer stem-like cells and high-grade oral squamous cell carcinoma. Clin. Cancer Res..

[54] Ren Z.H., Zhang C.P., Ji T. (2016). Expression of SOX2 in oral squamous cell carcinoma and the association with lymph node metastasis. Oncol. Lett..

[55] Habu N., Imanishi Y., Kameyama K., Shimoda M., Tokumaru Y., Sakamoto K., Fujii R., Shigetomi S., Otsuka K., Sato Y. (2015). Expression of Oct3/4 and Nanog in the head and neck squamous carcinoma cells and its clinical implications for delayed neck metastasis in stage I/II oral tongue squamous cell carcinoma. BMC Cancer.

[56] Islam F., Gopalan V., Wahab R., Smith R.A., Lam A.K. (2015). Cancer stem cells in oesophageal squamous cell carcinoma: Identification, prognostic and treatment perspectives. Crit. Rev. Oncol. Hematol..

[57] Patel M., Yang S. (2010). Advances in reprogramming somatic cells to induced pluripotent stem cells. Stem. Cell. Rev..

[58] Kamachi Y., Uchikawa M., Kondoh H. (2000). Pairing SOX off: With partners in the regulation of embryonic development. Trends Genet..

[59] Avilion A.A., Nicolis S.K., Pevny L.H., Perez L., Vivian N., Lovell-Badge R. (2003). Multipotent cell lineages in early mouse development depend on SOX2 function. Genes Dev..

[60] Gontan C., de Munck A., Vermeij M., Grosveld F., Tibboel D., Rottier R. (2008). Sox2 is important for two crucial processes in lung development: Branching morphogenesis and epithelial cell differentiation. Dev. Biol..

[61] Pesce M., Scholer H.R. (2001). Oct-4: Gatekeeper in the beginnings of mammalian development. Stem Cells.

[62] Reers S., Pfannerstill A.C., Maushagen R., Pries R., Wollenberg B. (2014). Stem cell profiling in head and neck cancer reveals an Oct-4 expressing subpopulation with properties of chemoresistance. Oral Oncol..

[63] Herr W., Cleary M.A. (1995). The POU domain: Versatility in transcriptional regulation by a flexible two-in-one DNA-binding domain. Gene Dev..

[64] Bourguignon L.Y., Earle C., Wong G., Spevak C.C., Krueger K. (2012). Stem cell marker (Nanog) and Stat-3 signaling promote MicroRNA-21 expression and chemoresistance in hyaluronan/CD44-activated head and neck squamous cell carcinoma cells. Oncogene.

[65] Bourguignon L.Y., Spevak C.C., Wong G., Xia W., Gilad E. (2009). Hyaluronan-CD44 interaction with protein kinase C(epsilon) promotes oncogenic signaling by the stem cell marker Nanog and the Production of microRNA-21, leading to down-regulation of the tumor suppressor protein PDCD4, anti-apoptosis, and chemotherapy resistance in breast tumor cells. J. Biol. Chem..

[66] Nakamura M., Nakatani K., Uzawa K., Ono K., Uesugi H., Ogawara K. (2005). Establishment and characterization of a cisplatin-resistant oral squamous cell carcinoma cell line, H-1R. Oncol. Rep..

[67] Hunter A.M., LaCasse E.C., Korneluk R.G. (2007). The inhibitors of apoptosis (IAPs) as cancer targets. Apoptosis.

[68] Gyrd-Hansen M., Meier P. (2010). IAPs: From caspase inhibitors to modulators of NF-κB, inflammation and cancer. Nat. Rev. Cancer.

[69] Bourguignon L.Y., Xia W., Wong G. (2009). Hyaluronan-mediated CD44 interaction with p300 and SIRT1 regulates β-catenin signaling and NFκB-specific transcription activity leading to MDR1 and Bcl-xL gene expression and chemoresistance in breast tumor cells. J. Biol. Chem..

[70] Clevers H. (2011). The cancer stem cell: Premises, promises and challenges. Nat. Med..

[71] Sherida C., Kishimoto H., Fuchs R., Mehrotra S., Bhat-Nakshatri P., Turner C., Goulet R., Badve S., Nakshatri H. (2006). CD44^+^/CD24^−^ breast cancer cells exhibit enhanced invasive properties: An early step necessary for metastasis. Breast Cancer Res..

[72] Marhaba R., Zoller M. (2004). CD44 in cancer progression: Adhesion, migration and growth regulation. J. Mol. Histol..

[73] Jiang W.G., Puntis M.C., Hallett M.B. (1994). Molecular and cellular basis of cancer invasion and metastasis: Implications for treatment. Br. J. Surg..

[74] Vasudevan S., Tong Y., Steitz J.A. (2007). Switching from repression to activation: MicroRNAs can up-regulate translation. Science.

[75] Chang S.S., Jiang W.W., Smith I., Poeta L.M., Begum S., Glazer C., Shan S., Westra W., Sidransky D., Califano J.A. (2008). MicroRNA alterations in head and neck squamous cell carcinoma. Int. J. Cancer.

[76] Bourguignon L.Y., Wong G., Xia W., Man M.Q., Holleran W.M., Elias P.M. (2013). Selective matrix (hyaluronan) interaction with CD44 and RhoGTPase signaling promotes keratinocyte functions and overcomes age-related epidermal dysfunction. J. Dermatol. Sci..

[77] Bourguignon L.Y. (2014). Matrix hyaluronan-activated CD44 signaling promotes keratinocyte activities and improves abnormal epidermal functions. Am. J. Pathol..

[78] Sasayama T., Nishihara M., Kondoh T., Hosoda K., Kohmura E. (2009). MicroRNA-10b is overexpressed in malignant glioma and associated with tumor invasive factors, uPAR and RhoC. Int. J. Cancer.

[79] Shilatifard A. (2006). Chromatin modifications by methylation and ubiquitination: Implications in the regulation of gene expression. Annu. Rev. Biochem..

[80] Ng H.H., Feng Q., Wang H., Erdjument-Bromage H., Tempst P., Zhang Y., Struhl K. (2002). Lysine methylation within the globular domain of histone H3 by Dot1 is important for telomeric silencing and Sir protein association. Genes Dev..

[81] Van Leeuwen F., Gafken P.R., Gottschling D.E. (2002). Dot1p modulates silencing in yeast by methylation of the nucleosome core. Cell.

[82] Feng Q., Wang H., Ng H.H., Erdjument-Bromage H., Tempst P., Struhl K., Zhang Y. (2002). Methylation of H3-lysine 79 is mediated by a new family of HMTases without a SET domain. Curr. Biol..

[83] San-Segundo P.A., Roeder G.S. (2000). Role for the silencing protein Dot1 in meiotic checkpoint control. Mol. Boil. Cell.

[84] Janzen C.J., Hake S.B., Lowell J.E., Cross G.A. (2006). Selective di- or trimethylation of histone H3 Lysine 76 by two DOT1 homologs is important for cell cycle regulation in *Trypanosoma brucei*. Mol. Cell.

[85] Bernt K.M., Zhu N., Sinha A.U., Vempati S., Faber J., Krivtsov A.V., Feng Z., Punt N., Daigle A., Bullinger L. (2011). MLL-rearranged leukemia is dependent on aberrant H3K79 methylation by DOT1L. Cancer Cell.

[86] Daigle S.R., Olhava E.J., Therkelsen C.A., Majer C.R., Sneeringer C.J., Song J., Johnston L.D., Scott M.P., Smith J.J., Xiao Y. (2011). Selective killing of mixed lineage leukemia cells by a potent small-molecule DOT1L inhibitor. Cancer Cell.

[87] Daigle S.R., Olhava E.J., Therkelsen C.A., Basavapathruni A., Jin L., Boriack-Sjodin P.A., Allain C.J., Klaus C.R., Raimondi A., Scott M.P. (2013). Potent inhibition of DOT1L as treatment of MLL-fusion leukemia. Blood.

[88] Kim W., Kim R., Park G., Park J.W., Kim J.E. (2012). Deficiency of H3K79 histone methyltransferase Dot1-like protein (DOT1L) inhibits cell proliferation. J. Biol. Chem..

[89] Ma L., Teruya-Feldstein J., Weinberg R.A. (2007). Tumour invasion and metastasis initiated by microRNA-10b in breast cancer. Nature.

